# Understanding and Modulating Immunity With Cell Reprogramming

**DOI:** 10.3389/fimmu.2019.02809

**Published:** 2019-12-11

**Authors:** Cristiana F. Pires, Fábio F. Rosa, Ilia Kurochkin, Carlos-Filipe Pereira

**Affiliations:** ^1^Cell Reprogramming in Hematopoiesis and Immunity Laboratory, Lund Stem Cell Center, Molecular Medicine and Gene Therapy, Lund University, Lund, Sweden; ^2^Wallenberg Center for Molecular Medicine, Lund University, Lund, Sweden; ^3^Center for Neuroscience and Cell Biology, University of Coimbra, Coimbra, Portugal; ^4^Center for Neurobiology and Brain Restoration, Skolkovo Institute of Science and Technology, Moscow, Russia

**Keywords:** cell fate reprogramming, transcription factor, hematopoiesis, dendritic cell, cancer immunotherapy, antigen presentation, transdifferentiation, regenerative medicine

## Abstract

Cell reprogramming concepts have been classically developed in the fields of developmental and stem cell biology and are currently being explored for regenerative medicine, given its potential to generate desired cell types for replacement therapy. Cell fate can be experimentally reversed or modified by enforced expression of lineage specific transcription factors leading to pluripotency or attainment of another somatic cell type identity. The possibility to reprogram fibroblasts into induced dendritic cells (DC) competent for antigen presentation creates a paradigm shift for understanding and modulating the immune system with direct cell reprogramming. PU.1, IRF8, and BATF3 were identified as sufficient and necessary to impose DC fate in unrelated cell types, taking advantage of Clec9a, a C-type lectin receptor with restricted expression in conventional DC type 1. The identification of such minimal gene regulatory networks helps to elucidate the molecular mechanisms governing development and lineage heterogeneity along the hematopoietic hierarchy. Furthermore, the generation of patient-tailored reprogrammed immune cells provides new and exciting tools for the expanding field of cancer immunotherapy. Here, we summarize cell reprogramming concepts and experimental approaches, review current knowledge at the intersection of cell reprogramming with hematopoiesis, and propose how cell fate engineering can be merged to immunology, opening new opportunities to understand the immune system in health and disease.

The immune system detects and eliminates invading pathogens, foreign particles, and tumor cells, in order to maintain homeostasis and protect from disease. Manipulation of the immune system has been explored since the eighteenth century with the discovery of the first vaccines to induce protective responses against infections [reviewed by (1)]. More recently, the ability to re-educate the patient's own immune system to recognize and eliminate tumor cells—cancer immunotherapy—has gained outstanding attention given the increased survival rates and long-lasting treatment results. For instance, antibodies targeting the inhibitory receptors CTLA-4 and PD-1 on the surface of T-cells unleash anti-tumor responses (2). The success of these immune checkpoint inhibitors, awarded with the 2018 Nobel Prize in Physiology or Medicine, has paved the way to the development of other immunotherapeutic strategies such as adoptive cell therapies. Chimeric antigen receptor (CAR) T cell approaches, which rely on *ex vivo* genetic engineering of autologous T cells, have also been recently approved for the treatment of hematologic malignancies (3). However, these cell-based approaches are still far from reaching their full potential due to limitations in obtaining sufficient cell numbers, expanding and manipulating immune cells *in vitro* and their functional compromised nature in some clinical settings. Improving these approaches will be of crucial importance to make cancer immunotherapy available and efficient for all patients, and not just to the minority that currently responds.

Cell fate reprogramming approaches have been classically developed to address questions of cell identity and epigenetic memory in the fields of developmental and stem cell biology. Given the potential to generate autologous cells for transplantation, such as functional cardiomyocytes and pancreatic β-cells, reprogramming is being explored for regenerative medicine to replace lost or damaged cells and tissues. The emergent ability to reprogram any human cell into desired hematopoietic cell types opens avenues to the discovery of new therapies for immune diseases. Here, we summarize cell reprogramming approaches, focus on the advances of reprogramming within the hematopoietic system, and envision how classic stem cell biology tools can be merged with immunology, generating new ideas for immunotherapeutic interventions.

## Cell Fate Reprogramming Concepts and Experimental Approaches

Cell reprogramming refers to the ability to redefine the identity of a cell by changing its epigenetic and transcriptional landscapes, reflected in the acquisition of new morphological, molecular, and functional features (4). These changes entail complete reversion of cell fate or modification of somatic cellular identity. Somatic cells can be reprogrammed to pluripotency, acquiring self-renewal and pluripotent features similar to embryonic stem cells (ESCs) (5, 6). Alternatively, lineage reprogramming involves conversion of specialized cells into a different somatic cell type without transiting through pluripotency (7). This process can occur directly (transdifferentiation or direct cell reprogramming) or progressing through an intermediate progenitor state that re-differentiates into different cell types.

**Figure 1:**
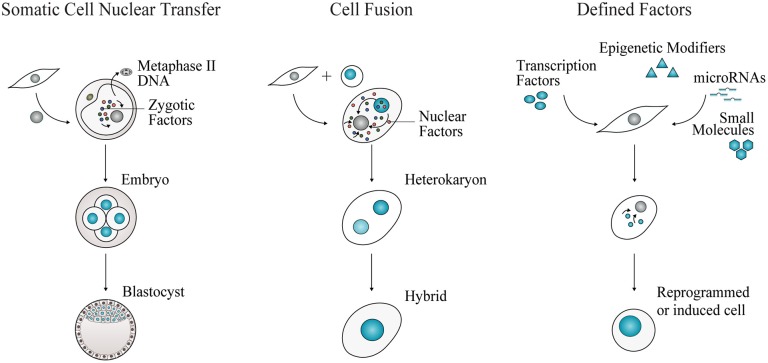
Experimental approaches for cell fate reprogramming. Nuclear transfer, cell fusion, and enforced expression of defined factors have revealed the plasticity of cell identity. Adult cell commitment can be experimentally reverted or modified by exposing a cell nucleus to unidentified or defined factors. In SCNT, a nucleus of an adult cell is transferred into an enucleated metaphase-II oocyte. The somatic cell nucleus is reprogrammed to totipotency by the action of zygotic factors. Cell fate can also be reverted or modified by cell fusion. Two cells are fused to generate a multinucleated heterokaryon, where nuclear factors shuttle across nuclei. Nuclear fusion gives rise to a tetraploid hybrid cell that is able to proliferate. Cell fate conversion can be accomplished by defined factors, including cell type-specific transcription factors, epigenetic modifiers, microRNAs and small molecules, acting in combination to impose pluripotency or alternative somatic cell identities.

Cell fate reprogramming can be achieved experimentally by three approaches, nuclear transfer, cell fusion, and enforced expression of transcription factors (Figure 1), bringing insights into the definition and regulation of cell identity. For more than a century, the theory of nuclear equivalence—specialized cells of metazoans possess a gene pool identical to that in the zygote nucleus—has been experimentally examined and debated (8, 9). Demonstrations of somatic cell reprogramming (10) have established that several types of differentiated cells indeed retain flexible lineage potential [reviewed by (11, 12)].

### Somatic Cell Nuclear Transfer

In somatic cell nuclear transfer (SCNT), the nucleus of a somatic cell is transplanted into an enucleated oocyte (Figure 1). In 1962, Gurdon generated fertile adult frogs after transferring nuclei from tadpole intestinal cells into irradiated oocytes (13). These results challenged the dogmatic view of cell differentiation. In vertebrates, differentiation of totipotent stem cells in the early embryo gives rise to progressively committed progenitors generating the constellation of highly specialized somatic cells that constitute an entirely new organism. For long, this process of cell specialization was considered an irreversible process, occurring with loss or permanent silencing of genetic information (8, 9).

Gurdon's seminal experiments showed for the first time that cell specialization involves changes in gene expression rather than gene content. These results demonstrated that nuclei of non-cycling and terminally differentiated cells contain the genes required to specify the development of an entire organism, a clone, upon reactivation by reprogramming. Later experiments by Gurdon, DiBerardino, and Hoffner further supported this conclusion by successfully cloning swimming tadpoles from adult keratinized skin and erythroid cell nuclei (14, 15). It took more than three decades for the first mammal to be cloned from an adult cell, Dolly the sheep (16), which was then followed by an array of different species. The unequivocal demonstration that a terminally differentiated cell can be reprogrammed to produce an adult cloned animal, and not due to a contaminating stem cell population, was provided when mice were cloned from terminally differentiated B and T cells carrying fully rearranged immunoglobulin alleles in all tissues (17). Human nuclear transfer-derived ESC lines have been generated from nuclei isolated from fetal and adult fibroblasts (18, 19). These cell lines can be used to generate desired cells (including hematopoietic cells) *in vitro* by differentiation, bypassing difficulties in obtaining ESC lines from human embryos (20). More recently, clones of higher primates were successfully generated with nuclei from macaque monkey's fetal fibroblasts (21). SCNT was combined with critical epigenetic modulation by injection of mRNA encoding H3K9me3 demethylase *Kdm4d* at one-cell stage that facilitates reactivation of pluripotency-associated genes.

Overall, SCNT has established the principle that the mechanisms underlying lineage restriction and cell identity are ultimately reversible, remarkably illustrated by the plasticity of different cell fates generated throughout development. SCNT has been explored as an alternative source of human ESCs for regenerative medicine applications, but a broad application is still hindered by the inefficiency of the process (22). In the future, cloning technology combined with genome editing may be used to generate improved disease models in non-human primates, to better mimic human pathophysiology and evaluate the efficacy of new immunotherapies (23).

### Cell Fusion

This experimental approach involves fusion of two or more cells to generate a single transient cellular entity containing more than one nucleus, termed heterokaryon (Figure 1). With time, the nuclei will fuse, giving rise to tetraploid hybrids able to proliferate. Early hybrid experiments uncovered the role of *trans*-acting repressors of gene expression, with the observation that some functional properties, such as melanin synthesis, ceased following fusion of different somatic cells (24). Silencing or loss of repressors after cell fusion was also observed by Harris' experiments providing the first indication of the existence of tumor suppressor genes (25). In hybrid cells derived from fusing malignant with non-cancerous cells, the malignant state was first suppressed. However, it re-emerged after proliferation, excluding the possibility of loss of oncogenes and rather validating the existence of negative regulators. Interestingly, in cell fusion experiments, the classic embryology principle of “phenotypic exclusion” or “discreteness” (26) was also observed, as hybrid cells display one or the other differentiated cell state, but not both concomitantly (27).

Data supporting activation of silent genes was later observed in heterokaryons of mouse muscle cells with human cells from the different germ layers (28, 29). The stable non-proliferative fusion product exhibited expression of human muscle genes, showing that gene expression repressed in differentiated cells could be reactivated by factors difused between nuclei. Different stoichiometry of fused cells or gene dosage was observed to influence reprogramming. Mixed species heterokaryons showing reactivation of human erythroid and hepatocyte-specific genes supported that the differentiated state of a somatic cell is highly plastic and dependent on continuous regulation (30, 31).

Cell fusion experiments also shed light on the mechanisms for imposing pluripotency in somatic cells. Tada and colleagues have demonstrated that somatic cells could be reprogrammed to pluripotency by generating hybrids with embryonic germ cells and ESCs (32, 33). Seeking to identify the factors that reset the genetic program of somatic cells, Do and Schöler reported that only karyoplasts, but not cytoplasts of ESCs, activated pluripotency genes upon cell fusion. This observation led to the conclusion that reprogramming ability resides in the nucleus (34). Experiments with mixed species heterokaryons contributed to a better understanding of the first phases of reprogramming to pluripotency. Reprogramming occurs fast and independently of nuclei fusion, which allowed the identification of early regulators of pluripotency, such as OCT4, Polycomb repressive complex (PRC), and activation-induced cytidine deaminase (AID) (35–37). More recently, hematopoietic stem and progenitor cells (HSPCs) were also shown to reprogram somatic cells to a progenitor phenotype upon cell fusion (38, 39).

### Defined Factors

Nuclear transfer and cell fusion experiments revealed that the nuclei of somatic cells can be reprogrammed to different cell fates by *trans*-acting factors. Together, they have prompted the identification of “nuclear” reprogramming factors. First reports of plasticity of cell fate mediated by defined factors (Figure 1) were made by Davis and colleagues: overexpression of the transcription factor MyoD in mouse fibroblasts induced conversion into the myogenic lineage (40). A sea change in the stem cell field was set in motion by Takahashi and Yamanaka with the identification of four transcription factors OCT4, SOX2, KLF4, and MYC, sufficient to induce reprogramming of mouse and human fibroblasts into pluripotency (5, 6). The resulting induced pluripotent stem cells (iPSCs) display self-renewal and pluripotency attributes similar to ESCs. These pioneering experiments revealed that a minimal transcription factor network is sufficient and necessary to erase the transcriptional and epigenetic identity of somatic cells, resetting it to a pluripotent state. It also demonstrated the stability of the reprogrammed cell state imposed by a small combination of transcription factors. Since then, several studies focused on elucidating the molecular mechanisms underlying the reprogramming process (41) as well as on increasing its efficiency by reprogramming different somatic cells, employing multiple combinations of factors and delivery methods [reviewed by (42, 43)]. Epigenetic modifiers, microRNAs, and small molecules have also been implicated in promoting the efficiency of reprogramming or by substituting at least partially transcription factor combinations [(44, 45) and reviewed by (46)].

Additionally, generation of human patient and disease-specific iPSCs circumvented ethical concerns associated with the use of human embryos. iPSCs derived from patients with neuromuscular and cardiac disorders generated somatic cells after differentiation that recapitulated phenotypic traits associated with the disease (47, 48). After these initial demonstrations, disease-specific iPSC-based models have been established and used to uncover molecular mechanisms associated with pathologic conditions as well as screening of new drugs and therapies (49). iPSC-derived cells have been also recently used to model cancer. Osteoblasts differentiated from iPSCs obtained from patients with Li–Fraumeni syndrome bearing germline p53 mutations recapitulated osteosarcoma features including tumorigenic ability upon subcutaneous injection in immunodeficient animals (50). More recently, iPSC-derived hematopoietic cells have also been used to model hematologic malignancies, providing insights into the clonal progression of acute myeloid leukemia and a platform to test stage-specific genetic and pharmacological interventions (51, 52). However, modeling of hematological disorders remains very challenging as protocols to differentiate hematopoietic cells from iPSCs fail to specify fully functional HSPCs and definitive hematopoiesis. In contrast, since microglia is thought to be originated from the primitive hematopoietic wave, iPSC-derived microglia cells have been generated as a model for neurological disorders (53, 54). iPSC technology has also been explored to generate mature somatic cells for regenerative medicine. Less than 10 years after the initial discovery, human iPSC-derived retinal cells were, for the first time, tested in clinical setting (55), followed by a panoply of other cell types currently being assayed for cardiac diseases, spinal atrophies, and others (56).

Direct cell reprogramming has also been achieved with defined factors. A diversity of somatic cell types including lineage-restricted progenitors and mature cells, such as neurons, cardiomyocytes, and hepatocytes, have been generated upon overexpression of lineage-specific transcription factors in other somatic cells [reviewed by (7)). Similarly to iPSC reprogramming, epigenetic regulators have been included in direct conversions, either by coordination with lineage-specific transcription factors (57) or by facilitating surpassing the epigenetic barriers that limit reprogramming (58). MicroRNAs improve the efficiency of the process or replace the combination of factors, even though not attaining the acquisition of fully functional features observed with transcription factors (59, 60). Small molecule-driven strategies have also been reported to increase the efficiency of conversion or to reduce the requirement for exogenous factors, facilitating clinical application of reprogrammed cells (61, 62). In some cases, pluripotency factors have also been combined with lineage-specific factors to promote plasticity in early phases of reprogramming (63). In such cases, the acquisition of a plastic state may be mediated by transit through the pluripotent state. This has been subsequently demonstrated (64, 65) and therefore not included in this review as *bona fide* direct cell reprogramming examples.

We propose here that in addition to its applications in autologous cell generation, direct cell reprogramming is a powerful discovery tool for understanding the underlying principles for imposing a gene regulatory network *de novo*. Conventional or conditional gene knock-out models have provided extensive knowledge about the role of single transcription factors at specific developmental points but little information of functional orchestration of cell identity by combinatorial action of transcription regulators. Moreover, compensation from closely related transcription factors often offer difficulties in drawing conclusions on the role of transcription factors through loss-of-function studies. On the other hand, knock-out experiments also provide useful information to guide the identification of candidate transcription factors able to instruct a specific lineage by reprogramming. However, this is not a universal feature for all instructive factors. For instance, HSPC specification is not severely impaired in mice lacking the AP1 transcription factor FOS (66, 67), but combinatorial overexpression of GATA2, GFI1B, and FOS is sufficient and necessary to induce a hemogenic program (68, 69). Thus, it can be incredibly valuable to apply a “learning by creating” based reprogramming approach. Indeed, interest has built to predict and identify such unique factors to induce every cell type in the organism (4, 70–72). Direct reprogramming distinguishes the instructive transcription factors (from all possible molecular switches impacting a specific cell lineage) that act in combination to initiate the cascade of events required to remodel and specify cell identity.

By bypassing pluripotency, direct cell reprogramming approaches offer an attractive alternative to generate adult somatic cells for transplantation. In contrast to the induction of pluripotency that requires activation of cell cycle progression (73), direct conversions toward mature states have been shown to occur without cell proliferation (74) and, in most cases, give rise to post-mitotic cells. Thus, in order to obtain sufficient number of cells for transplantation, expansion of the initial pool of somatic cells is necessary. Alternatively, strategies that give rise to multipotent stem cell populations have also been described and might enable large-scale use (68, 75–77). An extension of the direct cell reprogramming approach is to induce cell fate conversions *in vivo*. Conversions of cardiac fibroblasts into cardiomyocytes (78, 79) or astrocytes into neurons within the *in vivo* microenvironment have been reported (80), suggesting a potential application for heart or brain repair by reprogramming *in situ*.

In the hematopoietic system, several lineage reprogramming approaches have been reported, towards mature and progenitor cells with regenerative potential. We propose that lineage reprogramming can be expanded to generate the diversity of innate and adaptive immune cells, opening new and exciting avenues for the field of immunotherapy.

## Defined Factor-Mediated Reprogramming of Hematopoietic Cell Fates

**Figure 2:**
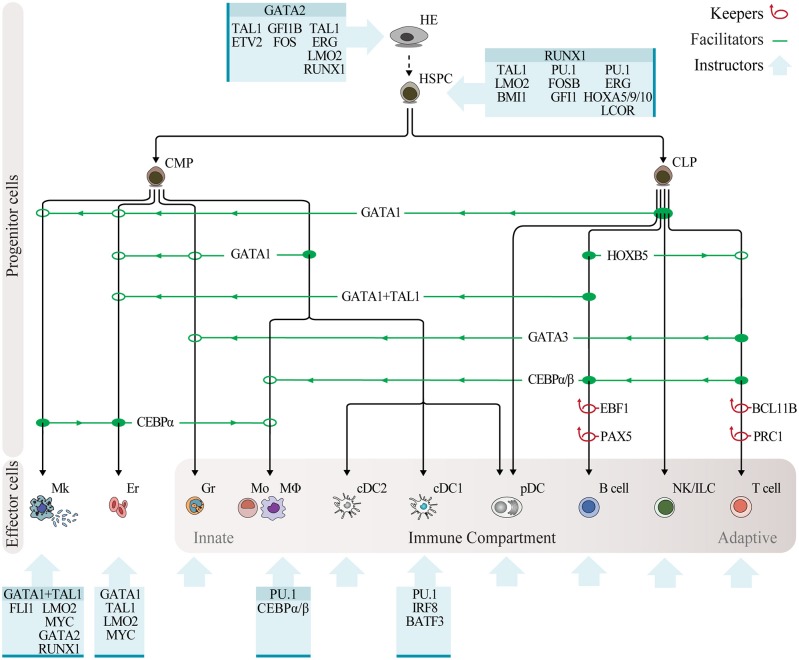
Programming hematopoietic cell fates with transcription factors. Overview of reprogramming approaches in the hematopoietic system, with transcription factors classified as keepers, facilitators, and instructors. Keepers are factors that maintain cell fate and repress alternative lineages, as determined by gene knockout experiments, and are depicted by red loop arrows. Transcription factor facilitators of lineage reprogramming within the hematopoietic system, as determined through enforced expression experiments, are indicated by green lines showing the lineage of origin (solid circle), direction of reprogramming (green arrows), and lineage outcome (open circle). Blue boxes depict combinatorial expression of instructive transcription factors leading to reprogramming of non-hematopoietic cells into hematopoietic cells. Instructive factors reported in multiple studies are highlighted on top. Abbreviations: HE, hemogenic endothelium; HSPC, hematopoietic stem and progenitor cell; CMP, common myeloid progenitor; CLP, common lymphoid progenitor; Mk, megakaryocyte; Er, erythrocyte; Gr, granulocyte; Mo, monocyte; MΦ, macrophage; cDC2, conventional dendritic cell type 2; cDC1, conventional dendritic cell type 1; pDC, plasmacytoid dendritic cell; NK, natural killer; ILC, innate lymphoid cell.

The blood system serves as a paradigm for understanding general concepts of cell commitment and regeneration (81). HSPCs balance self-renewal while maintaining the potential to generate the greatest diversity of lineages and cell fates a somatic stem cell can generate, including non-immune and all innate and adaptive immune cells (Figure 2). Phenotypic classification of hematopoietic cells performed by multicolor flow cytometry allows for prospective isolation of committed progenitors, which in combination with *in vitro* differentiation and transplantation systems have contributed to the definition of blood lineages (82). Recently, single-cell gene expression analysis and clonal fate tracing *in vivo* has made it possible to distinguish between hundreds of transcriptionally different cell types within the hematopoietic system (83). By combining these approaches, it is now possible to generate multi-dimensional, high-resolution images of the hematopoietic hierarchy (84). This well-defined system is ideal to uncover the molecular players and mechanisms that regulate lineage specification.

Loss-of-function studies with transgenic mouse models have uncovered the role of single transcription factors governing stage-specific hematopoietic specification. PU.1-deficient mice die during embryogenesis showing impairment of myeloid and lymphoid lineages, but normal megakaryocyte development (85). By contrast, disruption of the CCAAT enhancer binding protein alpha gene (C/EBPα) induces a selective block in differentiation of granulocytes and monocytes but normal numbers of mature lymphoid, erythroid and megakaryocytic cells (86, 87). Loss of function of GATA1 has shown its essential role in erythropoiesis and megakaryocytic development (88, 89). In the early 90s, Thomas Graf and colleagues pioneered overexpression strategies in hematopoiesis. It was reasonable to think that some hematopoietic factors would have similar properties to the myoblast-inducing MyoD. Overexpression of the transcription factor GATA1 in a multipotential chicken cell line induced expression of erythroid-megakaryocyte and eosinophil lineage markers with concomitant downregulation of monocytic markers (90). Alternatively, enforced expression of PU.1 suppressed GATA1 expression and promoted the exit from multipotency with commitment toward the granulocytic–monocytic lineage (91). Interestingly, even though the ablation of PU.1 affects all blood lineages, this overexpression experiment highlighted PU.1's instructive role for the myeloid compartment. These data, combined with other early lineage conversions within the hematopoietic system (92), opened new avenues to combine cell reprogramming with classic genetic studies to elucidate transcription factors' role in specifying hematopoiesis and investigate the plasticity of blood progenitor and mature cell fates.

Lineage reprogramming may occur by relieving the cell of the factor maintaining the lineage, by promoting lineage switch or by the action of combinations of transcription factors to overcome the transcriptional landscape of the somatic cell and impose a new program. Thus, we propose that reprogramming factors can be classified into three categories based on existing evidence: keepers, supporting the maintenance of cell fate and repressing alternative lineages; facilitators, facilitating lineage switch within the hematopoietic system; and instructors of cell fate, inducing hematopoietic cell identity when expressed in combination in non-hematopoietic cell types (Figure 2).

### Keepers

Converging evidence from the three main experimental approaches in reprogramming suggests that differentiated cells require continuous and active regulation to maintain their identity (93). Transcription factors with known crucial roles in maintaining the differentiated state represent keepers of cell fate. Their deletion leads to reversion into a plastic state with the potential to generate other cell types. The repression of alternative cell states can be reflected on lineage output after ablation.

A classic example of this dedifferentiation process is the selective ablation of *Pax5* in B cell progenitors, which leads to expression of non-B cell gene programs and potential to generate all blood lineages, including myeloid and T lymphocytic lineages but not B cells (94, 95). Upon transplantation in recipient mice, *Pax5*^−/−^ progenitors retain self-renewal and long-term reconstitution potential, but, contrarily to HSPCs, preferentially give rise to lymphoid progeny (96). *Pax5* deletion induces a reversion to uncommitted lymphoid progenitors, which still retain the ability to generate myeloid cells (97). A dedifferentiation process was also shown to occur in mature B cells (98). However, mice lacking *Pax5* in mature B cells developed aggressive lymphomas, hindering the prospective application of this approach to generate multilineage progenitors. More recently, a similar role as keeper of B cell fate was uncovered for EBF1 as its conditional deletion in committed B cell progenitors gives rise to innate lymphoid cells (ILCs) and T cells upon transfer into alymphoid mice (99). The synergy between EBF1 and PAX5 is illustrated by combined heterozygous deletions of *Pax5* and *Ebf1* resulting in the conversion into alternative T cell fates (100). A similar developmental checkpoint was identified for commitment of early T cell progenitors into the αβ T cell lineage. Loss of *Bcl11b* in double negative (DN) T cell precursors blocked their differentiation, by preventing the repression of genes associated with alternative cell fates, such as natural killer (NK), myeloid, dendritic cell (DC), and multipotent progenitors (101, 102). Indeed, loss of *Bcl11b* in progenitor or committed T cell populations leads to the generation of NK cells (103). BCL11B-deficient NK cells display morphology, surface expression, and cytotoxic activity against tumor cells *in vitro* and *in vivo*, resulting in the prevention of cancer progression in syngeneic mouse models.

Epigenetic regulators also play a role in cell fate maintenance during hematopoietic development. Inactivation of PRC1 components has been shown to induce conversion of T lineage progenitors into the B-cell fate (104). Deletion of PRC1 catalytic subunits, Ring1A/B, blocks mouse T cell development at early stage (DN3). These arrested progenitors gave rise to B cells after transplantation into immunodeficient mice. Interestingly, additional deletion of *Pax5* reverted the block in T cell differentiation, indicating that maintenance of T cell fate requires PRC-mediated epigenetic suppression of the B lineage program. Of note, *Bcl11b* expression was not increased after PRC1 inactivation, suggesting that Polycomb is not mechanistically synergistic with BCL11B in keeping T cell fate and probably justifying why NK cells are not induced. These combined data allow the establishment of a model of T cell specification in which BCL11B and PRC1 play a role in keeping cell identity by excluding different alternative cell fates (NK or B lineages).

### Facilitators

Evidence also supports the existence of factors that, when overexpressed, act as facilitators for changing cell lineages within the hematopoietic system. Experiments in transformed cell systems have motivated the evaluation of the plasticity of lineage commitment in primary hematopoietic cells. Overexpression of GATA1 in bipotent progenitors with ability to generate neutrophils and monocytes redirected cell fate to the erythrocytic, eosinophilic, and basophilic lineages (105). By performing clonal analysis, the authors have demonstrated true lineage switching induced by GATA1 as opposed to differential cell survival or selection of clones. Iwasaki and colleagues validated these results in granulocyte/monocyte progenitors (GMPs), and further showed that GATA1 converted less committed progenitors, such as HSPCs, common myeloid (CMPs), and lymphoid progenitors (CLPs) into megakaryocytes and erythrocytes (106). GATA1 specifically facilitated the megakaryocytic and erythrocytic lineage commitment while preventing alternative outcomes naturally generated by differentiation. Interestingly, expression of GATA1 could not facilitate conversion of mature hematopoietic cells into megakaryocytic/erythroid lineages. Using a hypothesis-driven approach, Sadahira and co-workers identified that SCL/TAL1 regulator needed to be combined with GATA1 to reprogram terminally differentiated B cells into the erythroid lineage (107). C/EBPα was shown to facilitate this type of reprogramming, possibly by its described ability to inhibit B cell fate keeper PAX5 and unlocking the commitment to the B cell lineage (108, 109).

Interestingly, C/EBPα alone has been implicated in inducing myeloid lineage conversion in fully differentiated B cells (108). Overexpression of C/EBPα or C/EBPβ leads to rapid and efficient reprogramming of B cells into macrophage-like cells, which acquire typical morphology, surface expression, and phagocytic function. This process occurs via silencing of *Pax5* and requires synergy with endogenous PU.1 to ensure activation of macrophage-specific genes. Similar conversion and requirement for endogenous PU.1 levels was observed for reprogramming committed T cell progenitors into macrophages (110). C/EBPα-induced conversion to macrophages has been further reported for megakaryocyte/erythrocyte progenitors, CLPs, and B-lineage progenitors (109, 111). A modified B cell line provided a uniquely efficient process with conversion toward macrophage fate occurring with 100% efficiency in 2–3 days (112), allowing dissection of mechanistic events. C/EBPα-induced conversion does not require cell division (113) and knocking-down p53 did not accelerate reprogramming kinetics, in contrast to what has been described in iPSCs reprogramming (114).

Another member of the GATA family of transcription factors, GATA3, has also been implicated as lineage switch facilitator. GATA3 is expressed in hematopoietic progenitors, thymocytes, and T cells and has been implicated as a regulator of early T cell commitment (115). It would be logical to think that GATA3 would facilitate or instruct T cell fate. Rothenberg and colleagues surprisingly found that overexpression of GATA3 in thymic T cell progenitors induced re-specification of T cells to the mast cell lineage (116). This lineage switch only occurred in the absence of Notch signaling at DN1 and DN2 stages of thymic development but not DN3, revealing that there is a developmental window necessary for plasticity. It also raises the question if these two seemingly disparate cell types, mast cells and T cells, are indeed developmentally closer than prior anticipated. Another possibility is functional redundancy with the homolog GATA2, highly expressed in mast cells and whose reporter has recently served to enrich in mast cells and progenitors differentiating from iPSCs (117).

HOXB5 is another example of a transcription factor that induces conversion into a cell type in which it is not expressed. HOXB5 is restricted to the top of the hematopoietic hierarchy, in HSPCs, but surprisingly converts B cell progenitors in T cells *in vivo* (118). The observed lineage switch does not seem to occur through dedifferentiation into uncommitted progenitor, as observed for *Pax5* and *Ebf1* deletion experiments. Instead, B cell progenitors are reprogrammed into an early T cell progenitor in the bone marrow that later matures in the thymus. *In vivo*, these cells give rise to fully functional CD4+ and CD8+ T cells with transcriptional profile, tissue distribution, and immune functionality similar to natural T cells. Ridell et al. also explored an *in vivo* approach to identify transcription factor combinations that could facilitate reversion of hematopoietic cell fates into HSPCs. The authors identified that transient expression of six transcription factors RUNX1T1, HLF, LMO2, PRDM5, PBX1, and ZFP37 in committed B cell progenitors followed by transplantation in irradiated recipient hosts imparts multilineage potential (119). Addition of N-MYC and MEIS1 to the reprogramming cocktail further increased the reprogramming efficiency and ability to induce long-term reconstitution potential. Committed myeloid progenitors and mature cells were also shown to be amenable to reprogramming, demonstrating that such combination of factors favors reversion into the HSPC fate from both the lymphoid and myeloid lineages. The conservation of this complex combination of factors in human cells needs to be verified. In both cases, the acquisition of T cell and HSPC fates requires that reprogramming occurs in the *in vivo* microenvironment. This dependence suggests that the identified factors are insufficient to impose the desired cell fate and additional intrinsic and extrinsic factors are necessary. Additionally, a careful mechanistic dissection of the lineage reprogramming process is challenging.

### Instructors

MyoD was initially identified to induce muscle reprogramming in fibroblasts but failed to generate multinucleated myotubes in a myriad of cell types (40, 120), suggesting that additional transcription factors were needed to induce reprogramming from developmental distant cell types. Similarly, GATA1 alone induces megakaryocyte cell fate from a multitude of hematopoietic progenitors (106), but requires the combined action of TAL1 and C/EBPα to convert fully committed B cells (107). More dramatic cell fate transitions for conversion of distant non-hematopoietic cell types into hematopoietic progenitors and mature cells require instructors of hematopoietic cell fate. This third category of transcription factors display ability to silence gene regulatory networks from the initial cell type and impose a completely different epigenetic and transcriptional program. Theoretically, once the combination of instructors of a particular cell fate is identified, every cell type in the organism could be reprogrammed to this fate. In addition, the action of this group of factors would be conserved between mouse and human, mirroring the induction of pluripotency. OCT4, SOX2, KLF4, and C-MYC were shown to induce pluripotency in mouse and human cells from an extensive range of cell types including fibroblasts, keratinocytes, erythroid progenitors, and T and B cells demonstrating the robustness of the network (5, 6).

Several groups have reported alternative strategies to generate HSPCs from different cell types of origin including fibroblasts, endothelial cells, and iPSCs. Interestingly, despite different starting cell types and culture conditions, some transcription factors are common between approaches, highlighting their key role as instructors of hematopoietic stem cell specification. The role of zinc-finger transcription factor GATA2 in instructing the emergence of HSPCs was clearly demonstrated in several reprogramming approaches (68, 69, 121, 122). In the embryo, HSPCs arise from specialized endothelial precursor cells with blood-forming potential, hemogenic endothelium (HE), through a process of endothelium-to-hematopoietic transition (EHT) (123, 124). GATA2 is expressed in HE cells in the embryo and null GATA2 animals die at E11.5 at the time of HSPC generation (125). We have shown the role of GATA2 in instructing the emergence of HE. Using a human CD34 reporter system, we have identified GATA2, GFI1B, and FOS as the minimal transcription factor network to induce a hemogenic program in mouse fibroblasts (68). These three factors induce a dynamic, multi-stage hemogenic process that progresses through an endothelial-like state, recapitulating developmental hematopoiesis (68). Lineage reprogramming allowed capturing *in vitro* an intermediate whose phenotype was then confirmed to occur during embryogenesis (126). A similar conceptual approach has been recently applied to the isolation of neural plate border stem cells, further supporting the value of direct reprogramming to inform development (127). GATA2, GFI1B, and FOS combination was also recently shown to give rise to long-term repopulating HSPCs when expressed in iPSC-derived teratomas *in vivo* (122), and to induce hemogenesis in human fetal and adult fibroblasts (69). These data support their role as instructors of hematopoietic origin from multiple species and cell types. Other studies also demonstrated the importance of GATA2: forced expression of GATA2 combined with ETV2 or TAL1 in human pluripotent stem cells induced “forward programming” into human HE cells, which upon differentiation give rise to pan-myeloid hematopoietic cells or erythroid and megakaryocytic cells, respectively (128). In another study, GATA2 and TAL1 factors gave rise to HE cells, when combined with ERG, LMO2, and RUNX1 (121). Overexpression of the five factors in mouse fibroblasts induced stably expandable HE when co-cultured with stromal cells and short-term engraftment upon transplantation. In this system, multilineage potential with generation of lymphoid cells required further ablation of p53.

Several reprogramming reports have also highlighted the role of RUNX1 in instructing hematopoietic multipotency. *Runx1*^−/−^ embryos lack intra-aortic hematopoietic clusters and HSPCs (129), while specific ablation of *Runx1* in endothelium cells impairs EHT and establishment of definitive hematopoiesis (130). RUNX1 expression is essential for HSPC specification in the embryo (131), but is no longer required for maintaining HSPCs in the adult (132). Overexpression of RUNX1, TAL1, LMO2, and BMI1, combined with extracellular BMP and MEK signaling, in mouse fibroblasts was shown to induce HSPCs with ability to engraft immunodeficient mice and generate myeloerythroid and B lymphoid progeny (133). Alternatively, mouse and human endothelial cells were shown to give rise to hematopoietic progenitors upon expression of RUNX1, SPI1, GFI1, and FOSB transcription factors (134). An *in vitro* engineered vascular supportive system was required for the induction of multipotent progenitors, which engrafted primary and secondary immunodeficient animals, generating all hematopoietic lineages except T cells. By further refining the system, Rafii et al. have subsequently shown that transient expression of the four factors in mouse endothelial cells, combined with vascular-niche-derived angiocrine factors, converts them into HSPCs with long-term self-renewal and ability to differentiate into multilineage progeny, including reconstitution of T cell adaptive immune function (135). It is interesting to note that AP1 paralogs, namely FOSB and FOS, as well as the repressors GFI1 and GFI1B have been identified as instructive factors of HSPC identity by Pereira et al. (68) and Sandler et al. (134). This points to the potential functional redundancy between these factor combinations, especially considering that endothelial cells express high levels of GATA2, providing a potential explanation for these differences in factor requirements.

Human HSPCs with long-term engraftment properties have also been recently generated from iPSCs using a cocktail including RUNX1. Sugimura et al. first used a small-molecule and hematopoietic cytokine cocktail to induce HE cells from human pluripotent cells (136), which were then used to screen combinations of transcription factors that impart long-term multilineage reconstitution properties. RUNX1, PU.1, ERG, HOXA5, HOXA9, HOXA10, and LCOR generated engraftable cells that reconstituted myeloid, B, and T cells in primary and secondary recipients. Taken together, these reports suggest that additional external signals are necessary to promote adequate maturation of the reprogrammed HSPCs. It will be interesting to assess if additional transcription factors can be added to the cocktail mimicking the instructive role of the “niche”.

Transcription factors that work as instructors of mature hematopoietic cell fates upon expression in non-related cell types have also been reported. Overexpression of GATA1, TAL1, LMO2, and c-MYC in mouse and human fibroblasts induces direct conversion to erythroid progenitors with gene expression profile resembling primitive erythroblasts (137). Interestingly, addition of KLF1 or MYB promoted expression of adult globin pattern, despite not inducing a switch from primitive to a definitive gene expression program. Given the developmental proximity of the megakaryocyte and erythrocyte lineages, the authors then screened for additional factors that could tilt the reprogramming toward the megakaryocyte cell fate. GATA2 and RUNX1 in combination with the transcription factor core (GATA1, TAL1, LMO2, and c-MYC) efficiently converted mouse and human fibroblasts into megakaryocyte-like progenitors showing polyploidies and polylobulated nuclei that give rise to platelets upon *in vitro* and *in vivo* maturation (138). Alternatively, instructive factors with ability to guide differentiation of human pluripotent cells into the megakaryocyte lineage have also been described (139). GATA1 and TAL1 megakaryocyte-instructors combined with FLI1 induce forward programming of proliferative megakaryocyte progenitors that differentiate and mature *in vitro*. It is interesting to notice that both GATA1 and TAL1 have also been previously implicated as facilitators of megakaryocyte/erythrocyte cell lineages in conversions within the hematopoietic system (105–107), but once combined with additional factors, instruct these lineages from unrelated cell types, such as fibroblasts or pluripotent cells.

Similarly, C/EBPα alone is not sufficient to convert fibroblasts into macrophages, despite its described ability to convert megakaryocyte/erythrocyte progenitors, CLPs, T and B lineage progenitors, and fully mature B cells. Building on the observation that endogenous PU.1 levels were required for reprogramming committed T cell progenitors (110), Graf and co-workers combined C/EBPα with PU.1 to induce conversion of a fibroblast cell line into macrophage-like cells (140). PU.1 and C/EBPα silence fibroblast genes while up-regulating macrophage transcriptional signature, driving the acquisition of phagocytic ability. Alternatively, overexpression of PU.1 alone induce conversion of adult neural stem cells, but not mature cells, into monocytes (141). Given that C/EBPβ is expressed in neural stem cells and plays a crucial role in the maintenance of the self-renewal properties (142), it is interesting to ask whether reprogramming is mediated by cooperation with endogenous C/EBPβ expression.

Taken together, facilitators may work as instructors when combined with the right partners. Direct or indirect interaction with additional factors might be required to impose a given cell fate in developmentally distant cells, and such factors may be available in closely related lineages. These additional partners might also facilitate chromatin engagement of the facilitator. This cooperative action may be sufficient to silence the gene regulatory network of any initial cell type and impose a new transcriptional and epigenetic identity. Moreover, it is still to be determined if transcription factors described as keepers of specific cell fate might work as instructors and in what conditions, for example, by repressing the identity of alternative fates or the identity of the initial cell. It would be interesting to evaluate the reprogramming capacity of keepers of lymphoid identity in combination with other factors to instruct lymphoid commitment.

Of note, several combinations of three factors have been identified as instructive of pluripotent (143), neural (144), or hematopoietic progenitor cell fates (68). The rule of three is applied in everyday life in popular sayings, slogans, comedy and politicians' speeches, to make communication more engaging as three is the smallest number required to create a pattern recognized by the human brain. The rule of three, *omne trium perfectum* (every set of three is complete), also seems to apply to direct cell reprogramming approaches mediated by instructive factors (145). Every set of three may represent a minimal cooperative regulatory assembly (146) to instruct the core of cell identity. This core may need to be combined with external cues in order to fully recapitulate their natural counterparts or accessory factors to modulate independent properties in the cell (such as MYC and the proposed role in cell proliferation) (143).

Induction of hematopoietic cell fates, such as HSPCs and megakaryocyte/erythrocyte cell fates from unrelated cell types, has mainly been accomplished for cells with therapeutic potential for regenerative medicine. However, generation of mature hematopoietic cells with the ability to modulate immune responses remains largely unexplored. Recently, our group provided evidence that antigen-presenting DCs can be induced from mouse and human fibroblasts by a combination of three instructive transcription factors (147). In the next sections, we will dwell on our strategy to identify such factors, how this approach informs development and lineage heterogeneity, as well as how it can be expanded to other immune cell types and lead to the development of powerful immunotherapies.

## Reprogramming Antigen-Presenting Dendritic Cells

DCs are known as professional antigen-presenting cells given their remarkable ability to link innate and adaptive immunity. Acting as immune sentinels throughout the body, DCs patrol the tissues looking for foreign bodies and pathogens, engulf and process them, integrating environmental signals and feeding those to the appropriate effector cells of the innate and adaptive immune systems (148). DCs are originated from HSPCs through monocyte dendritic cell progenitors (MDPs) that further differentiate in the bone marrow by losing the ability to give rise to the monocytic lineage. Common dendritic cell progenitors (CDPs) give rise to subset-primed committed progenitors that migrate into the blood and seed the tissues, originating different mature DC subsets categorized according to transcription factor dependence, surface marker expression, tissue localization, and functional specialization (149). Plasmacytoid dendritic cells (pDCs) produce type I interferons, crucial for mounting anti-viral responses. Classical or conventional dendritic cells (cDCs) can be further divided into two major subsets: type 1 (cDC1), which excel on cross-presentation of exogenous antigens on MHC-I, eliciting CD8+ T cytotoxic responses, and type 2 (cDC2), which govern type 2 and 3 immune responses against parasites and extracellular bacteria, activating ILC2/3 and Th2/17 cells (150).

**Figure 3:**
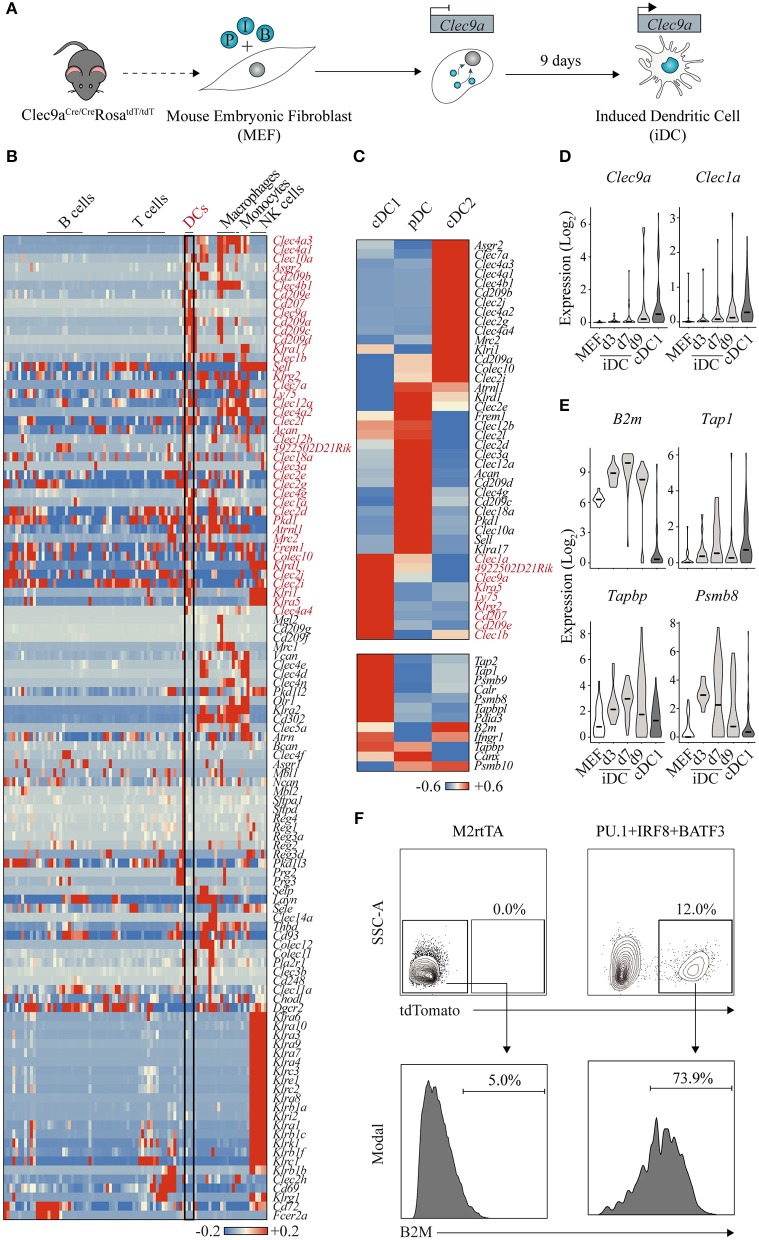
Clec9a-tdTomato reporter allows the identification of dendritic cell-instructive transcription factors. **(A)** Using double transgenic mouse embryonic fibroblasts (MEF) expressing Cre recombinase under the control of *Clec9a* promoter and Rosa26-lox-stop-tdTomato, we identified PU.1 (P), IRF8 (I), and BATF3 (B) to program induced dendritic cells (iDC) affiliated with the conventional DC type 1 (cDC1) fate. **(B)** Heat map showing gene expression of C-type lectin receptors across hematopoietic cell lineages (GSE127267). Forty-two receptors expressed in DCs are highlighted in red. DC, dendritic cell; NK, natural killer. **(C)** Heat maps showing differential gene expression of C-type lectin receptors (upper panel), where nine genes enriched in cDC1s are highlighted in red, and genes associated with antigen cross-presentation (bottom panel) in mouse cDC1, cDC2, and plasmacytoid DCs (pDC) (GSE103618). Red indicates increased expression, whereas blue indicates decreased expression over the mean. **(D)** Expression levels of *Clec9a* and *Clec1a* and **(E)** *B2m, Tap1, Tapbp*, and *Psmb8* during DC reprogramming at days 3 (d3), 7 (d7), and 9 (d9). Log values of census counts are shown. Horizontal lines correspond to median values. **(F)** Flow cytometry analysis of B2M surface expression in tdTomato-positive cells generated by transduction with PU.1, IRF8, and BATF3 after 9 days. MEF transduced with M2rtTA were included as control. Adapted from Rosa et al. (147).

We hypothesized that lineage reprogramming could be employed to generate antigen-presenting DCs from mouse and human fibroblasts (Figure 3A) (147). In order to identify instructive factors to impose DC fate, we first compiled a list of 18 candidate factors whose expression is restricted to the DC lineage and enriched during DC ontogeny. Importantly, loss of function of the candidate factors was associated with impaired DC development or function. To test the DC-inducing ability of the candidate factors, we relied on mouse embryonic fibroblasts bearing a Clec9a reporter system.

CLEC9A, also named DNGR-1 (DC, NK-lectin receptor-1), is a member of the C (Ca^2+^-dependent)-type lectin receptors (CLRs), a superfamily composed of more than 100 surface receptors that play a pivotal role in DC ability of screening tissues for damage or dying cells, microbial or viral presence (Figure 3B) (151). After ligand binding, CLRs modulate gene transcription, promote microbicidal activity or activate the endocytic and phagocytic machinery. Among the immune cell compartment, CLRs are expressed differently; for example, almost all members of the *Klra* gene family, encoding for Ly49 type II CLR receptors, are specifically expressed in the NK cell population (152). Regarding DCs, 42 CLRs are also differentially expressed across the three subsets (Figure 3C). DCIR2 and LY49Q, encoded by *Clec4a4* and *Klra17* genes, are specifically expressed in cDC2 and pDC subsets and have been associated with MHC-II presentation and IFN type I secretion, respectively (153, 154). Several CLRs are specific to the cDC1 subset. For some, like *Clec1a* and *Klrg2*, not much is known and there is still no identified ligand. In contrast, Langerin/*Cd207* binds sugars on the surface of bacteria, fungi, and HIV-1 as well as self-ligands exposed by dead cells, while DEC-205/CD205/*Ly75* recognizes apoptotic cells and oxidized lipids (155, 156). Both receptors have been shown to trigger internalization and processing of cargo for antigen presentation on MHC molecules. Importantly, antigen targeting through these receptors allows efficient delivery to cross-presentation on cDC1s, driving Th1 and CD8+ T cell immunity (157). In parallel with expression of these CLRs, cDC1s are also characterized by the expression of genes of the cross presentation pathway, namely, processing of the antigens by *Psmb8, Psmb9*, and *Psmb10* immunoproteasome components, *Tap1* and *Tap2* transporter proteins, *Tapbp* and *Pdia3* chaperones required for proper loading of the antigen, and components of the MHC class I heterodimer, such as *B2m* (Figure 3C, bottom panel) (158).

CLEC9A receptor is also highly expressed in cDC1s and contributes to cDC1-specific functional attributes. Mouse CLEC9A is expressed in high levels in splenic CD8a+ and tissue-resident CD103+ cDC1s, and at lower extent in pDCs (147). In a lineage tracing model, Clec9a^Cre/Cre^Rosa26^tdTomato/tdTomato^ (Clec9a-tdTomato) reporter has been shown to label the DC population and their committed progeny starting at the CDP level (159). Recently, CLEC9A has also been identified as a discriminative marker for the human cross-presenting CD141+ DC1 subset, the human equivalent to the mouse cDC1 subset (160, 161). Functionally, CLEC9A receptor has been shown to recognize ligands exposed on the surface of necrotic dead cells, triggering endocytosis and promoting cross-presentation of dead cell-associated antigens (162, 163).

We hypothesized that fibroblasts carrying a Clec9a-tdTomato reporter system would be ideal to allow the identification of instructive transcription factors that induce DC fate and functional properties (147). By screening 18 candidates, we have identified PU.1, IRF8, and BATF3 (PIB) transcription factors as the minimal transcription factor network sufficient and necessary to induce Clec9a-tdTomato reporter activation and impose DC fate in mouse fibroblasts. Induced DCs (iDCs) acquire DC morphology with cytoplasmic protusions and dendrites, and surface expression of DC markers and antigen presentation machinery (MHC-II, MHC-I, CD40, CD80, and CD86). Interestingly, we have detected the expression of XCR-1 and CD103 cDC1-specific surface markers, but failed to detect robust expression of cDC2 and pDC markers. Transcriptional profiling of the iDC population further confirmed the affiliation specifically to the cDC1 subset, and not a mixed population of the different DC subsets. iDC reprogramming is a direct process with no progenitor intermediate, and cDC1 transcriptional reprogramming is achieved after 9 days of forced expression of PIB. In addition to *Clec9a*, iDCs also express other cDC1-enriched CLRs, such as *Clec1a* (Figure 3D). Functionally, iDCs acquire responsiveness to toll-like receptor 3 (TLR3) and TLR4 stimuli secreting inflammatory cytokines and ability to engulf antigens including dead cells. Remarkably, iDCs cross-present extracellular antigens and induce antigen-specific CD8+ T cell responses (147). Concordantly, we have observed that during the reprogramming process, iDCs acquire expression of cross-presentation genes, such as *B2m, Tap1, Tapbp*, and *Psmb8* (Figure 3E). At the protein level, the reprogrammed population (labeled by the Clec9a-driven tdTomato expression) activates B2M expression at the cell surface, reflecting the expression of MHC-I complexes at the cell membrane required for antigen presentation (Figure 3F). We have further shown that PIB forced expression reprograms human embryonic and adult fibroblasts into human DCs that express DC1 markers, such as CD141+ and CLEC9A+ (147), opening avenues for modulating immune responses in humans with direct cell reprogramming. The efficiency of human DC1 reprogramming was however low, and future studies need to be centered on optimizing reprogramming conditions in order to guarantee feasibility of a potential application. In addition, thorough *in vivo* functional characterization of the mouse iDCs' properties will provide supportive evidence on the equivalence to endogenous *bona fide* cDC1 cells. These results will pave the way for translation of cell reprogramming approaches as therapeutic tools in the context of immunotherapy. It would also be interesting to test whether variations of the reprogramming combinations of factors would promote the induction of other subsets of DCs. Here, Clec genes, such as *Dcir2* and *Ly49Q*, would be valuable to report cDC2 and pDC-lineage reprogramming, combined with established cDC reporters such as Zbtb46-GFP (164).

## Creating is Understanding

### Direct Cell Reprogramming Informs the Establishment of a New Gene Regulatory Network

The identification of PU.1, IRF8, and BATF3 as instructors of cDC1 cell fate in fibroblasts provides a platform to study how these transcription factors impose cDC1 identity. Mechanistic studies to elucidate reprogramming events have been extensively applied to the induction of pluripotency [reviewed by (41)] and to a lesser extent to direct cell reprogramming approaches, such as conversion to neuronal fate (165, 166).

In the hematopoietic system, a limited number of studies dwell into the molecular mechanisms of direct reprogramming and how reprogramming factors engage chromatin. In the HOXB5-driven conversion of B cell precursors into the T cell lineage, HOXB5 targets have been characterized by ChIP-seq 3 days after retroviral transduction (118). The authors have shown that HOXB5 binds directly to important B and T cell regulators, such as *Ebf1* and *Bcl11a*, which are downregulated and upregulated, respectively. HOXB5 also directly targeted genes encoding chromatin-modifying enzymes, such as *Kmt2a*. However, this reprogramming system requires an *in vivo* step and complete silencing of B cell specific regulators was only observed after homing to the bone marrow and maturation in the thymus. Thus, it is hard to draw conclusions on the direct molecular mechanisms required for the acquisition of early thymic progenitor identity.

*In vitro* reprogramming approaches provide a system for detailed mechanistic dissection. During C/EBPα-mediated conversion to monocyte cell fate, C/EBPα was shown to establish a myeloid expression program in pre-B cells by binding two types of myeloid enhancers (167). Pre-existing enhancers, broadly active throughout the hematopoietic lineage including B cells, are occupied by PU.1 driving C/EBPα targeting. In contrast, C/EBPα acts as a “pioneer factor” in *de novo* enhancers, making them accessible for PU.1 recruitment. However, it remains to be investigated whether a similar combined instructive action of PU.1 and C/EBPα occurs from fibroblasts to macrophages.

**Figure 4:**
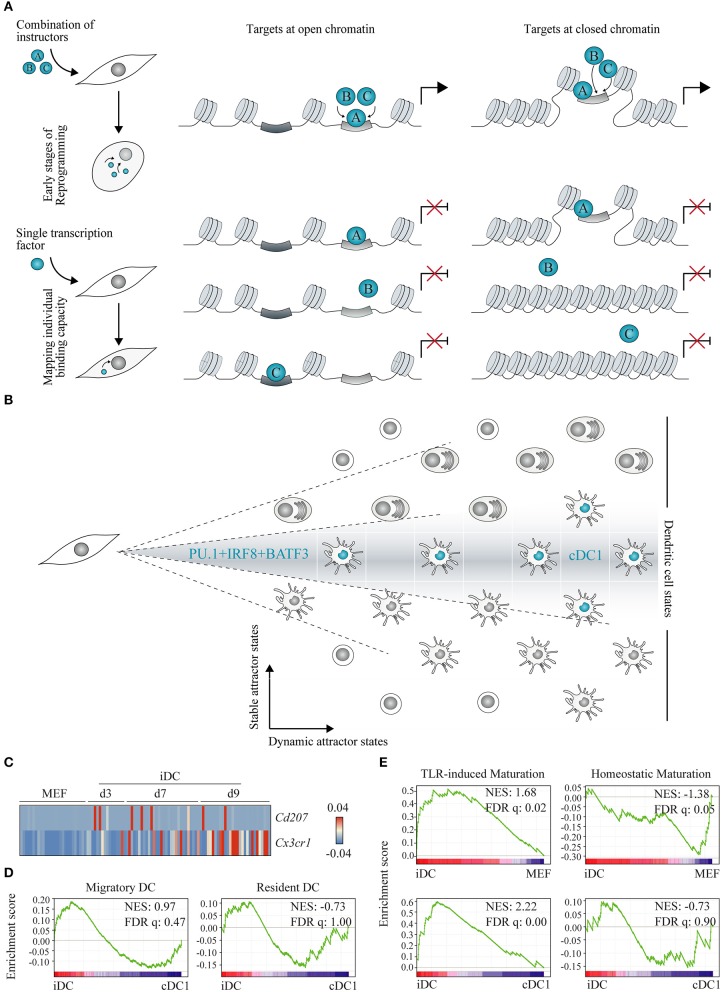
Understanding gene regulatory networks and lineage heterogeneity with cell reprogramming. **(A)** Two models have been proposed for instructive transcription factor (represented as A, B and C) binding to chromatin: (1) cooperative binding among multiple transcription factors at open chromatin regions leading to a gradual increase in site accessibility (left panel), and (2) pioneer transcription factors, as a unique class of transcriptional regulators with the capacity to bind nucleosomal DNA (closed chromatin, right panel). Experimentally, these models can be interrogated at the onset of lineage reprogramming in fibroblasts by expressing instructive transcription factors in combination (top panel) or individually (bottom panel) followed by genome location studies. **(B)** Combined expression of PU.1, IRF8, and BATF3 in fibroblasts generates conventional dendritic cells type 1 (cDC1)-like cells capturing part of the DC lineage heterogeneity. DC cell states are depicted as positions in a matrix and cDC1 are highlighted in blue. We hypothesize that direct lineage reprogramming offers an exciting way to distinguish stable from dynamic DC attractor states according to their dependence on transcription factor combination and stoichiometry (dashed lines). **(C)** Heat map showing single cell gene expression of *Cd207* and *Cx3cr1* during reprogramming of mouse embryonic fibroblasts (MEF) to induced DCs (iDC) at days 3 (d3), 7 (d7), and 9 (d9) (GSE103618). **(D)** Gene set enrichment analysis between d9 iDC and splenic cDC1 was performed using migratory and resident DC gene sets (168), and **(E)** between d9 iDC and MEF, and d9 iDC and cDC1 (GSE103618), with TLR-induced or homeostatic maturation gene sets (169). NES, normalized enrichment score; FDR q, false discovery rate *q* value. Adapted from Rosa et al. (147).

Direct cell reprogramming strategies are particularly suited for dissection of how instructive transcription factors interact with chromatin to initiate reprogramming (Figure 4A). Profiling genomic engagement sites of transcription factors, when expressed in combination and individually at early time points, helps elucidating how they engage chromatin (Figure 4A). This approach, when combined with chromatin accessibility and transcriptional profiling, contributes to clarify how instructive factors silence the epigenetic and transcriptional signature of the initial cell type and activate the new gene regulatory network. In general, two models have been proposed. Transcription factors bind open chromatin sites, facilitating the engagement of other factors to the same site. This cooperative binding property may depend on a direct interaction between the transcription factors or through DNA elements (170). Alternatively, some transcription factors, often named “pioneer factors”, have the ability to access nucleosomal DNA and closed chromatin sites, increasing the accessibility of a target site to other reprogramming factors, chromatin-binding proteins and transcriptional machinery, initiating transcriptional reprogramming (165, 171).

Recently, we have used this strategy to dissect the molecular mechanisms underlying hemogenic induction (69). Genome-wide binding profiles of GATA2, GFI1B, and FOS were profiled by ChIP-seq of human fibroblasts 2 days after transduction with the three factors combined or each factor individually. This approach uncovered that GATA2 displayed independent targeting capacity, binding to similar chromatin sites when expressed alone or in combination (depicted in Figure 4A, left panel). In contrast, GFI1B alone binds to GFI and AP1 motifs, but when in combination with GATA2 and FOS, is recruited to additional sites, including GATA motifs. Interestingly, we observed that binding occurred at open chromatin regions flanking upstream transcription start sites and active enhancers. This mechanism of accessing available somatic enhancers that gradually leads to nuclear remodeling has also been described in reprogramming to pluripotency (170) (Figure 4A, left panel), and it is opposed to the model of direct engagement of repressed chromatin (depicted in Figure 4A, right panel). Our data further indicate that GATA2 and GFI1B physically interact and cooperatively repress expression of fibroblast genes and activate hematopoietic regulators, such as RUNX1. FOS showed a small number of targets in either condition, suggesting a limited access to chromatin during initial stages of reprogramming. It will be interesting to further dissect its chromatin binding and instructive role at later stages of reprogramming.

Genomic location studies in primary stage-specific hematopoietic progenitors have been limited by high cell numbers required to allow ChIP-seq analysis of transcription factors (172). Some studies have employed immortalized cell lines (173, 174) or hematopoietic progenitors obtained by differentiation of pluripotent cells, which normally promote embryonic over definitive hematopoiesis (175). Thus, direct reprogramming studies represent a good alternative to obtain a large number of initial cells and map factor binding. Additionally, overexpression of combinations of a limited number of instructive transcription factors facilitates the analysis of potential cooperative interactions and their role in specifying the acquisition of hematopoietic cell fates, as each factor individually is not sufficient to induce reprogramming but the combined efforts are (Figure 4A). Starting from fibroblasts also represents a major advantage as chromatin accessibility and histone maps are readily accessible from the ENCODE project (176). Transcription factor targets can be easily compared with a myriad of chromatin marks to shed light on the requirements of transcription factor targeting to chromatin (165). In contrast, by profiling natural hematopoietic cells in which several lineage-specific transcription factors are co-expressed, it is harder to identify which factors and interactions are crucial for the acquisition of cell fate rather than their maintenance.

In the future, it will be interesting to apply a similar approach to elucidate how PU.1, IRF8, and BATF3 interact with each other and chromatin to induce silencing of fibroblast genes and activation of cDC1 transcriptional signature. PU.1 has been classically classified as a pioneer factor, given its ability to identify DNA-binding sites even if partially occluded by nucleosomes. PU.1 is able to reposition the nucleosomes, allowing recruitment of other tissue-specific gene regulatory elements during development. This “nucleation” ability of PU.1 has been described in macrophages (177), B cells (178), and early T cell development (179). However, ability to bind “first” to chromatin has been described to still require additional co-factors or DNA binding proteins to further attract the machineries required to change chromatin landscape and modulate gene expression (178, 179). Accordingly, in neuronal reprogramming, ASCL1 pioneer binding correlated with a “trivalent” chromatin signature of H3K4me1, H3K27ac, and H3K9me3, which fail to predict all ASCL1 occupancy sites, suggesting that additional factors play a role (165). It will be interesting to understand if PU.1 repositions nucleosomes opening the sites for IRF8 and BATF3 during DC reprogramming, or if a cooperative effort at open chromatin is in place.

Another pioneer candidate is IRF8. IRF8 has been shown to modulate massive, cell-specific chromatin changes in pDC enhancers (180). IRF8 is essential for the establishment of monocyte and DC-specific enhancers during MDP differentiation (181). Notably, in *Irf8*^−/−^ progenitors, all H3K4me1, H3K27ac, and PU.1 signals in IRF8-bound enhancers were severely reduced, suggesting that IRF8 directly promotes enhancer priming and might be necessary for PU.1 binding.

In addition to interaction with ETS transcription factors at composite ETS-IRF sites, IRF8 can also be recruited to interferon-response DNA elements through interaction with AP1 factors, such as BATF3, and cooperatively mediate gene expression in cDC1s during pathogen infection (182, 183). During cDC1 development, it was previously proposed that BATF3 binding to a +32 kb *Irf8* enhancer containing AP1-IRF consensus sites creates an auto-regulatory loop to maintain high levels of IRF8 after commitment to cDC1 lineage (184). Recently, the deletion of this enhancer has shown to be essential to direct the transition from pre-cDC1 to cDC1 by supporting IRF8 expression (185). Another IRF8 enhancer was shown to be crucial to guide CDP to pre-cDC1 transition before BATF3-controlled enhancer becomes active (185).

Another described feature of PU.1 instructive role during myeloid specification is its dose dependence, rather than an On/Off switch. Graded levels of PU.1, which depend on the co-expression of other lineage-specific factors, are important to define the outcome at developmental decision nodes. High levels of PU.1 have been proposed to act as a rheostat in driving specification toward CDPs and preventing generation of neutrophilic lineage by remodeling IRF8 chromatin landscape (186, 187). Moreover, IRF8 and PU.1 doses have been implicated in imposing DC-lineage bias in human HSPCs (188). In our system, a high PU.1/IRF8 ratio is critical for induction of cDC1s (147).

Direct cell reprogramming provides a tractable system to address instructive transcription factor interaction and stoichiometry requirements for cell fate specification. It will be interesting to investigate which enhancers are targeted during activation of endogenous IRF8 expression observed during DC reprogramming (147). Insights from DC reprogramming mechanisms will also contribute to clarify how these instructive factors cooperate to impose cDC1 fate during development. Given the difficulties in isolating rare populations of DCs from mouse and human, current studies have been mainly done with *in vitro* bone marrow-derived cells, which mostly lack the functional properties of natural DCs and are composed of mixed populations of DC subsets. Generation of mouse knock-out systems to elucidate the mechanisms regulating the expression of lineage-specific transcription factors, as has been done for IRF8 enhancers (185, 189), is laborious and can be complemented with direct cell reprogramming.

### Direct Cell Reprogramming Informs Heterogeneity of Cell Fates

DCs constitute a remarkably heterogeneous hematopoietic lineage in respect to developmental origin, subset affiliation, anatomic location, maturation states, and functional properties (149, 190). A complex interaction of transcription factors, chromatin regulatory elements, and gene transcription profiles governs the specification of each cell lineage. The diversity of cell states and how they are specified can be conceptualized as “attractor” states in the epigenetic landscape proposed by Waddington (191–193). Physicists view attractor states as higher-order states of equilibrium. This concept can be transposed to biological systems as low-energy cellular states that correspond to transcriptional and epigenetic signatures. In theory, there is a finite number of attractor states given that some combinations of the different sources of heterogeneity give rise to energetically unbalanced states.

Given the identification of DC instructive factors (147), we hypothesize that direct cell reprogramming can be used to inform DC heterogeneity and categorize attractor states as stable or dynamic (Figure 4B). Stable attractor states are dependent on the nature of each transcription factor combination and their stoichiometry. In contrast, multiple dynamic attractor states are possible for the same combination of instructive factors, reflecting additional levels of regulation.

In the neuronal field, several combinations of factors, mostly containing ASCL1, have been described to induce neurons with mixed phenotype or favor dopaminergic, motor, or sensory neurons [reviewed by (194)]. Recently, additional combinations of instructive factors have been identified to induce a diverse population of neurons, each displaying unique transcriptional profiles and functional features (195). A similar approach by substituting transcription factors would be applicable to inform the diversity of DC stable attractor states.

In our study, notwithstanding the cDC1 affiliation, iDCs represent a heterogeneous population of cells with a subset of day 9 iDCs displaying a transcriptional profile reminiscent of IFN stimulation (147). By interrogating the transcriptomic profile of single reprogrammed DCs, it is possible to shed light on the heterogeneity of the population. Natural cDC1s have been reported as heterogeneous as well. CD8α+CD207+ DCs have been shown to be critical for the typical cDC1 functional features of cross-presenting antigens to CD8+ T cells and secreting IL12 (157, 196). Splenic CX3CR1+CD8α+ DCs with pDC-like features, such as E2-2 expression and immunoglobulin rearrangements, have also been described (197) and affiliated to the cDC1 subset (164). Interestingly, the expression of *Cd207* and *Cx3cr1* is mutually exclusive in iDCs, with no induced cells activating the expression of both markers (Figure 4C) and indicating that PU.1, IRF8, and BATF3 reprogramming captures this degree of heterogeneity inside the cDC1 compartment. Moreover, a unique transcriptional signature has been described for tissue migratory cDCs, independently of their tissue and cellular origin (168). Gene set enrichment analysis showed moderate enrichment of the migratory signature in iDCs, while resident gene set was more enriched on splenic cDC1 cells (Figure 4D), suggesting that iDCs are heterogeneous in terms of migratory and resident profiles. *Cd207*+ and *Cx3cr1*+ expression, as well as migratory and resident functional attributes, represent dynamic attractor states that are reflected in the population of iDCs.

Immature cDCs undergo homeostatic maturation, activating a tolerogenic profile important for the establishment of central and peripheral tolerance, or immunogenic maturation driven by TLR stimuli (169). Interestingly, the signature associated with immunogenic maturation is highly enriched in iDCs at day 9 when compared with initial fibroblasts and freshly isolated splenic cDC1 (Figure 4E). It is particularly remarkable that PU.1, IRF8, and BATF3 overexpression is sufficient to activate such gene signature even without TLR engagement, possibly indicating that this functional trait constitutes a stable attractor state, a consequence of the combination of transcription factors. Indeed, by profiling TLR-stimulated bone marrow DCs, it has been shown that chromatin marks at promoters and enhancers and a large number of transcription factors displayed very little dynamics throughout the stimulatory response and were mostly established during DC development (198).

In addition, it would be interesting to address whether PU.1, IRF8, and BATF3 have the ability to “rejuvenate” cells, as observed for reprogramming to pluripotency, or in contrast retain the aged phenotype as induced neurons do (199). Given the panoply of DC attractor states, it will be interesting to characterize the output of direct cell reprogramming experiments using instructive factors combined with extrinsic signals.

## Direct Cell Reprogramming for Immunotherapy: Future Applications

Understanding cell fate plasticity has opened the way for multiple applications, ranging from disease modeling to drug development and transplantation. The main advantage of cell reprogramming relies on generating patient-tailored cells from available cell sources with the same genetic content. However, for a successful cell-therapy application, these induced cells, such as neurons, are required to integrate in the damaged tissue, survive, and exert their functional properties long term. These requirements place the bar high for *in vitro* generated cells for regenerative medicine. In the case of DCs, long-term engraftment and function might not be necessary as soon as a robust memory response is mounted. Merging direct cell reprogramming with immunotherapy is particularly attractive given the current success of harnessing the immune system to tackle cancer.

**Figure 5:**
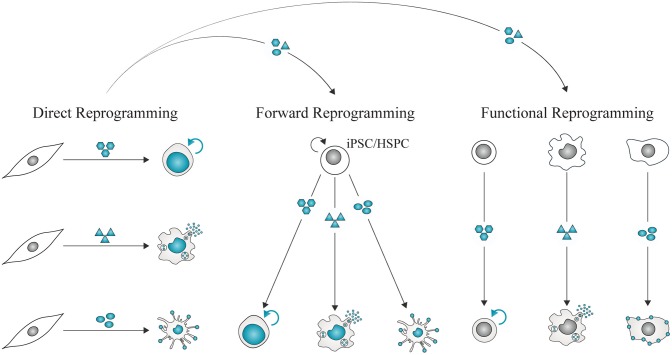
Direct reprogramming for immune-therapeutic applications. Direct lineage reprogramming allows the identification of instructive combinations of factors required to impose immune cell fates in unrelated somatic cells such as fibroblasts. These combinations can be repurposed to derive immune cells from expandable cell sources, such as iPSCs or hematopoietic stem and progenitor cells (HSPC). Forward reprogramming mediated by instructors represents a strategy to generate large quantities of homogeneous populations of immune cells for therapeutic applications. It may also be possible to impose functional reprogramming, or confer immune functional properties with instructors while retaining some features of the starting cell type.

Due to the professional antigen-presenting capacity, DCs have been explored for tumor vaccination (200). Advances have been made for DC-based immunotherapy, but the clinical outcome has been inconsistent, which may be associated with limiting number of hematopoietic progenitors cells, often compromised in cancer patients, and low efficiency of antigen presentation by autologous monocyte-derived DCs (201). In particular, cDC1s, or the human equivalent DC1 cells, are essential for inducing T cytotoxic responses and tumor clearance (160, 202). However, on one hand, current *in vitro* differentiation protocols usually give rise to mixed populations of DC subsets, and on the other hand, human DC1s are an extremely rare population, hindering their isolation from peripheral blood. Adult fibroblasts could offer a viable alternative to generate human *bona fide* DC1 by direct reprogramming. In contrast to reprogrammed stem cells and progenitors that raise safety concerns for transplantation purposes, reprogrammed DCs are appealing as they stop dividing (147) and DC lifespan is usually short (from 1 to 10 days) (203). Moreover, in the context of immunotherapy, iDCs may be used to elicit antigen-specific immune responses and no prolonged functionality or engraftment would be required, since long-lasting effect on adaptive immunity would rely on T cell memory (Figure 5). During DC reprogramming, we have observed an activation signature of TLR-induced maturation, opening avenues for modulating functional properties of reprogrammed cells independently of triggering TLR receptors.

Reprogrammed immune cells may also be valuable to model immune disease. Using clustered regularly interspaced short palindromic repeats (CRISPR)-Cas9, human iPSCs were modified with the HIV-protective CCR5Δ32 mutation to render differentiated monocytes resistant to HIV infection (204). Modeling immune dysfunction with DCs has been hindered by difficulties in DC subset availability. Thus, generating iDCs from patients with primary immune dysfunction with unknown cause or associated with reduced DC numbers or function (205), may provide a tractable way to attribute defects in antigen presentation to clinical scenarios of immune dysfunction.

In addition to DCs, the generation of other mature immune cells by reprogramming is very attractive for cancer immunotherapy. Generation of functional T cells by reprogramming could represent a source of cells to be combined with expression of chimeric antigen receptors (CAR) (206). CAR T cell therapies for B cell malignancies are already on the market, generated by modification of peripheral blood T cells from each patient. However, alternative sources of T cells are being explored to surpass difficulties in obtaining sufficient number of naïve CD8+ T cells from lymphopenic patients. In these patients, T cells are often exhausted, so alternative sources such as differentiating T cells from iPSCs are being explored (207). These systems are still under development to recapitulate a fully functional T cell and require extensive cell culture steps with cytokines or artificial thymic organoids (208).

In addition, NK cells and macrophages modified with CARs are also being explored for cancer immunotherapy. CAR-NK cells have attractive advantages compared with CAR T cells, as they express their native receptors and are still capable of recognizing and killing cancer cells even if they downregulate the CAR-targeted antigen. Moreover, NK cells have increased ability to infiltrate solid tumors, have a better safety profile not causing cytokine release syndrome, and do not require strict HLA matching (209). CAR-NK cells generated from peripheral blood, umbilical cord blood, and one irradiated lymphoma cell line have already been tested in clinical trials, and more recently, CAR-NK cells from human iPSC are also being explored as an alternative (210). Interestingly, iPSC-derived NK cells not carrying a CAR are currently being tested in the clinical setting, constituting the first example of immune cells generated by cell fate reprogramming to reach patients.

Macrophages modified with CARs to direct the engulfment of specific antigens have also been generated, showing increased ability to phagocyte cancer cells (211). It would be interesting to address if macrophages generated by direct cell reprogramming would also acquire such properties upon CAR modification. Despite being less established, B cells can also activate an antigen presentation phenotype and play a role in driving anti-tumor responses upon engineering with chimeric IL-2 and TGF-β (212) or IL-4 and GM-CSF (213). Neutrophils were shown to mediate anti-tumor responses and tumor regression triggered by a tyrosine kinase inhibitor (214). In summary, immune cells spanning the full spectra of innate and adaptive immune compartments are now starting to be uncovered as attractive mediators for cancer immunotherapy. Understanding their individual instructive factors and minimal gene regulatory networks by direct reprogramming (Figure 5) will generate valuable tools for immunotherapy.

In addition to cancer, CAR T cells are also being tested for the treatment of infectious diseases. CAR T cells have been modified to target human immunodeficiency virus (HIV)-infected cells (215) and early clinical trials confirmed the safety and feasibility of the approach but failed to induce sustained reduction on the viral load (216). These studies are now re-emerging as the improvements in CAR design done for cancer are being translated and adapted to fight HIV infection [reviewed by (217)]. Recently, multispecific anti-HIV CAR T cells targeting different portions of the HIV envelope protein were shown to control HIV infection in a humanized mouse model (218). iPSC-derived NK cells, unmodified or carrying HIV-targeted chimeric receptor, were also developed and were shown to suppress HIV replication and CD4+ T cell infection *in vitro* and *in vivo* (219, 220). Proof of principle for CAR T cells targeting fungal antigens was also accomplished (221), opening opportunities to design CAR T cell-based therapies for other infectious diseases (222).

On the other side of the spectrum, it has been proposed to use immune cell-based strategies to control exacerbated immune responses in the context of immune-mediated diseases, such as autoimmunity and allergy, and to promote tolerance to transplanted tissues and organs. For example, antigen-specific Foxp3+ regulatory T cells have been shown to suppress auto-immune diabetes and graft rejection (223, 224). CAR-modified regulatory T cells have been shown to control auto-immune responses in mouse models of colitis and multiple sclerosis and promote graft tolerance (225–228). More recently, CAR T cells targeting CD19 were also shown to promote elimination of auto-antibody-producing B cells and control lupus symptoms (229). In the future, regulatory immune cells generated by direct reprogramming, like regulatory T cells and DCs, will contribute to the development of such therapies. Moreover, identifying the requirements necessary to instruct a regulatory versus stimulatory immune fate will contribute to a better understanding of the delicate balance governing immunity and tolerance.

In addition to using induced cells in cell-based therapies *per se*, the identification of minimal combinations of instructive transcription factors provides additional therapeutic opportunities. Reprogramming can be merged to differentiation strategies, as the combination of instructive factors can be used to guide the differentiation from iPSCs or HSPCs—forward programming (Figure 5). This strategy has been shown to rapidly and efficiently convert human PSCs into neurons, skeletal myocytes, and oligodendrocytes (230). Within the hematopoietic system, transcription factor-mediated forward programming of pluripotent cells has been shown to induce human HE cells and proliferative megakaryocyte progenitors (128, 139). In the context of immunotherapy, it will be particularly attractive to explore this system to robustly generate high numbers of homogeneous populations of immune cells from expandable stem cells. Addressing whether PU.1, IRF8, and BATF3 expression in iPSCs or HSPCs generates cross-presenting DC1 cells would provide proof of principle to apply forward programming strategies to generate immune cells.

We also hypothesize that combinations of instructive transcription factors can be used to induce functional reprogramming (Figure 5). In some cases, an incomplete conversion might be desirable, with induction of functional properties combined with components of the initial cells. A classic example of such reprogramming process was provided by early cell fusion experiments. Antibody-secreting B cells were fused to myeloma cells generating immortalized hybridomas that produce antibodies with predefined specificity, and still constitute the standard procedure to obtain monoclonal antibodies (231). Cell fusion has also been explored as an alternative to target tumor-associated antigens to DCs and promote their immunogenicity as cancer vaccines. Hybrids of cancer cells and bone marrow-derived DCs have been shown to present tumor-associated antigens on MHC I and MHC II molecules, driving anti-tumor immune responses (232).

With the advent of reprogramming strategies mediated by defined factors, it will be interesting to investigate whether functional reprogramming can be used to induce therapeutically attractive properties. It has been proposed that iPSC reprogramming could be used to rejuvenate exhausted T cells [reviewed by (233)]. In fact, recent reports highlighted the role of intrinsic T cell factors (such as transcription of genes associated with memory T cells) determining clinical response to CAR T cell therapy (234). In addition, loss of the epigenetic enzyme TET2 was shown to reduce T cell differentiation and promote expansion of potent memory T cells (235). Thus, it is conceivable that reprogramming factors instructing specifically the memory phenotype could be directly used to rejuvenate T cell exhaustion and promote clinical efficacy of CAR T cell strategies.

In the context of cancer, several studies showed that conversion of the tumor-associated macrophages from the immunosuppressive M2 phenotype to the immunostimulatory M1 polarization state would also be therapeutically valuable [reviewed by (236)]. This conversion has been attempted using TLR agonists, interfering with signaling and metabolic components, single transcription factors, and epigenetic modulators. Such manipulations have demonstrated prevention of tumor immunosuppression and a synergistic effect with immune checkpoint inhibitors to improve anti-tumor responses. It would be relevant to explore direct cell reprogramming to elucidate the molecular determinants governing macrophage polarization opening avenues to a more robust manipulation of these attractor states.

In the future, it would be interesting to evaluate PU.1, IRF8, and BATF3 efficacy in inducing antigen presentation directly in tumor cells. Induced antigen presentation may bypass limitations of current cancer immunotherapies such as tumor cell heterogeneity, immune evasion, and neoantigen identification. Independent lines of evidence support the concept that cDC1s are not only important for cross-priming T-cell after migration into the lymph nodes, but their presence within the tumor microenvironment is necessary for effector T-cell recruitment, T cell-mediated tumor rejection, and increased patient survival (237–239). Thus, forcing a cDC1 phenotype by reprogramming *in vivo* within the tumor is therapeutically attractive, not only to promote tumor-associated antigen presentation but also to recruit effector T-cells for tumor clearance.

It has been a decade full of excitement seeing nuclear reprogramming findings transiting into clinical applications in regenerative medicine. Reprogramming to pluripotency and direct reprogramming of alternative somatic cells have emerged as two central paradigms for manipulating cell fate. The place that the immune system occupies in these paradigms has however received less attention. In the framework of the emerging synthetic biology era, we postulate that direct cell reprogramming will capture the plasticity and dynamics of a multitude of immune cell states at single-cell resolution. Consequently, this will allow us to redefine our understanding of immune cell lineages and their regulatory circuits to design new strategies for the expanding field of immunotherapy.

## Author Contributions

CP, FR, and C-FP conceptualized this manuscript. CP, FR, and IK analyzed mRNAseq datasets. FR prepared the figures. CP and C-FP wrote the manuscript.

### Conflict of Interest

The authors declare that the research was conducted in the absence of any commercial or financial relationships that could be construed as a potential conflict of interest.
